# Genetic and Epigenetic Alterations Associated With Human Prenatal Tobacco and Environmental Tobacco Smoke Exposure: Protocol for a Systematic Evidence Map

**DOI:** 10.3389/phrs.2025.1606742

**Published:** 2025-07-14

**Authors:** Ana Teresa Reis, Joana Madureira, João Paulo Teixeira, Carla Costa

**Affiliations:** ^1^ Environmental Health Department, National Institute of Health Doutor Ricardo Jorge, Porto, Portugal; ^2^ EPIUnit – Instituto de Saúde Pública, Universidade do Porto, Porto, Portugal; ^3^ Laboratório para a Investigação Integrativa e Translacional em Saúde Populacional (ITR), Porto, Portugal

**Keywords:** human prenatal exposure, genotoxicity, epigenetics, tobacco, environmental tobacco smoke

## Abstract

**Objectives:**

This protocol outlines the development of a systematic evidence map (SEM) on genetic and epigenetic alterations associated with human prenatal tobacco exposure. The SEM will identify and synthetize epidemiological data on periconceptional and prenatal tobacco exposure associated with genetic (e.g., DNA damage) and epigenetic (e.g., DNA methylation) outcomes. Furthermore, it will describe the available evidence, highlight knowledge clusters, and identify gaps for future research.

**Methods:**

Bibliographic databases and grey literature sources will be searched, complemented by reference mining. Predefined inclusion and exclusion criteria will guide study inclusion. Data extraction will include population, exposure, comparator, outcome, funding, study design, confounding factors, and statistical methods. Summarization will include a narrative review, graphical visualization, and an interactive, queryable and open-access table.

**Results:**

A pilot study assessed the protocol’s feasibility, testing key components, including screening, data extraction, and eligibility criteria. Findings confirmed that the methodology is workable and reliable.

**Conclusion:**

This protocol supports a rigorous, reproducible, transparent SEM, aligned with international standards. The comprehensive mapping will support research prioritization and inform public health policies targeting maternal and child health.

## Introduction

It is well documented that tobacco smoking or exposure to environmental tobacco smoke (ETS) during pregnancy has numerous adverse health effects on both mother and fetus, such as pre-eclampsia [1], ectopic pregnancy [2], miscarriage [3, 4], placental abruption and placenta previa [5, 6], fetal growth restriction, low birth weight, body length and head circumference [7–10], preterm delivery [11, 12], stillbirth [13] and birth defects [14]. Maternal smoking during pregnancy is also linked to increased risk of sudden infant death syndrome [15], as well as long-term consequences on the offspring from infancy [16, 17] to adulthood [18].

Over the past half century, owing to public health awareness campaigns and the enforcement of strict tobacco control policies, smoking prevalence has declined by 27.2% (26.0%–28.3%) for men since 1990, and by 37.9% (35.3%–40.1%) for women [19], but the prevalence of smoking pregnant women remains elevated, particularly in some high-income countries, such as Ireland (38.4%), Uruguay (29.7%), Spain (26.0%), and Denmark (25.2%) [20]. In low- and middle-income countries (LMICs), the prevalence of tobacco use among pregnant women is currently estimated to be low (a prevalence of 0.51%–0.90% was determined by Shukla et al. [21], and of 1.8%–3.6% by Caleyachetty et al. [22]), but exposure to ETS is a known health problem [23]; both are likely to increase in coming years since the number of smokers in LMICs is rising [24, 25]. Hence, and considering that smoking during pregnancy is a leading modifiable risk factor for poor birth outcomes, this topic remains an important public health concern generally addressed by different international agencies, such as the World Health Organization [26], the US Department of Health and Human Services [27], or the European Institute of Women’s Health [28].

The underlying biological mechanisms for the diverse effects of maternal smoking or ETS exposure during pregnancy are of particular interest as they may provide important insights into a preventable health risk [29]. Increasing evidence from human epidemiological studies shows that short- and long-term adverse health effects (measured at different life stages) associated with prenatal tobacco exposure may be mediated by genetic and epigenetic alterations (e.g., [30–34]).

The genotoxic potential of prenatal tobacco exposure has been confirmed by the higher frequencies of DNA strand breaks [35, 36], such as double-strand DNA breaks identified in placenta samples [37], micronuclei in cord blood [38], increased in chromosomal instability in umbilical cord blood [39], and oxidative damage in the placenta [40] and cord blood [41, 42]. Concerning epigenetic effects, smoking or exposure to ETS during pregnancy has been associated with altered DNA methylation in placental tissue [43, 44] and cord blood [34, 43–45], that can still be detected in exposed offspring for many years [30, 31, 46–49].

Given the pace at which knowledge appears to be growing, it is becoming increasingly challenging to keep track of existing evidence in the genetic and epigenetic epidemiological field associated with human prenatal tobacco and ETS exposure, underscoring the need for structured approaches, such as systematic reviews and systematic evidence maps (SEMs) to organize and synthesize the expanding body of literature, identify research gaps, and support evidence-based decision-making and policy development.

Among these approaches, SEMs have emerged as a particularly valuable tool to provide a broad overview of existing evidence by identifying patterns, trends, and gaps in the literature. Unlike systematic reviews, they do not assess the quality or risk of bias of individual studies but instead organize and present the evidence base in a structured, often visual format, to support research scoping and decision-making [50, 51]. This approach helps guide future research by revealing well-studied areas and highlighting evidence gaps.

A key advantage of SEMs is their inclusivity—they incorporate a wide range of study designs and qualities to ensure comprehensive coverage of the available evidence [52]. To support transparency and enable a general assessment of the robustness of included studies, SEMs can extract and report descriptive characteristics such as study design, sample size, the use of control groups, and the types of exposure and outcome measurements. While this does not constitute a formal risk of bias assessment, it provides end users with contextual information that may help interpret the strength and consistency of the mapped evidence. This broadness makes SEMs especially useful for informing funding priorities and shaping future systematic reviews. By enhancing transparency and accessibility, SEMs are increasingly being applied across diverse fields such as public health, environmental science, and toxicology [50, 51].

To begin the preparation of this SEM, an extensive search using the terms “pregnancy,” “tobacco,” “genetic,” “epigenetic,” and “systematic review” or “evidence map” conducted on PubMed,^1^ CINAHL,^2^ Epistemonikos,^3^ PROSPERO,^4^ Open Science Framework Registry,^5^ and Zenodo^6^ databases, commonly used for registration and publication of systematic reviews or evidence maps, showed that existing systematic approaches covering tobacco and/or ETS exposure during pregnancy predominantly focus on its relation with birth outcomes or specific diseases, such as respiratory and cardiac in the offspring (e.g., [53, 54]). In contrast, genetic and epigenetic mechanisms remain largely overlooked, with only one systematic review examining alterations in DNA methylation and dysregulation of miRNA expression after maternal smoking during pregnancy [55]. More importantly, no systematic evidence map covering the full scope of genetic or epigenetic outcomes of prenatal exposure to tobacco and/or ETS was found.

Hence, we herein describe the protocol to conduct a systematic evidence map aimed at identifying and compiling the available evidence on this topic, following a consistent, objective, rigorous, unbiased and transparent approach [50].

### Objectives of the Protocol

The primary objective of this protocol is to provide a detailed, pre-defined methodological plan for conducting a SEM on genetic and epigenetic alterations associated with human prenatal tobacco and environmental tobacco smoke exposure. By doing so, this protocol aims to ensure that the evidence mapping process is transparent, reproducible, and methodologically sound.

Specifically, this protocol aims to:

1. Define the scope and research question(s) of the SEM, including the population, intervention/exposure, comparators, and outcomes of interest.
2. Detail the search strategy, including information sources (bibliographic databases and grey literature), and search terms, to ensure comprehensiveness and reproducibility.
3. Describe the benchmarking process used to evaluate the comprehensiveness of the search strategy by comparing retrieved results with a curated list of relevant publications.
4. Establish clear eligibility and exclusion criteria for the selection of studies, ensuring consistency and objectivity in screening and inclusion.
5. Outline procedures for screening, data extraction, and coding, including the use of tools, and quality control measures (e.g., double screening).
6. Specify the planned approach to data presentation and visualization, and any interactive components.
7. Promote transparency and reduce bias in the SEM by making the procedure publicly available before the evidence mapping is carried out.
8. Conduct a pilot study to test and refine key components of the SEM workflow, including the application of inclusion and exclusion criteria, the structure and usability of data extraction templates, and the clarity and consistency of categorization schemes.
9. Facilitate replication and updates of the SEM in the future by providing a complete methodological record.

By making this protocol available in advance of conducting the SEM, we aim to enhance the reliability and credibility of the mapping results and contribute to best practices in evidence synthesis, while achieving our proposed goals for the SEM (described below).

### Objectives of the Systematic Evidence Map

Focusing on the immediate and the long-term genetic and epigenetic effects in the offspring resulting from prenatal human exposure to tobacco (tobacco use and/or ETS exposure), this SEM aims to describe methodological strategies used across included studies, to identify knowledge clusters and evidence gaps, and highlight emerging research questions and future research priorities, while assessing whether there is sufficient information on any specific topic worth pursuing a full systematic review.

The framework to this SEM, established as Population, Exposure, Comparator and Outcome (PECO) statement, is presented in Table 1.

**TABLE 1:** Population of interest, Exposure, Comparator, and Outcomes (PECO statement) of this systematic evidence map (Worldwide, 2023).

Population	Pregnant women and their offspring (different lifestages may be considered: newborns, children, adolescents, adults and elder)
Exposure	*In utero* exposure to active smoking and/or environmental tobacco exposure (related to cigarettes)
Comparator	Population not exposed (*in utero*) or exposed to lower levels of tobacco and/or environmental tobacco smoke than the exposed subjects
Outcome	Any endpoint on genetic and epigenetic alterations measured in the analysed population. Genetic changes include alterations in the DNA structure and sequence, and epigenetic changes comprise DNA methylation, histone modifications, and miRNA profiling

Data gathered in this SEM will be able to clarify the following questions:

• Regarding Population
  a. Which populations have been assessed (offspring in different lifestages, e.g., newborns, children, and adults)?
  b. Where has most data been collected (countries)?
• Regarding Exposure
  a. Which type of exposure has been considered (tobacco use and/or ETS exposure)?
  b. Which timings of prenatal exposure have been studied (whole pregnancy or some particular period)?
  c. How were exposure levels assessed (direct or indirect methods)?
  d. If by human biomonitoring, which matrices and assays have been used?
  e. If by questionnaire, which information was collected?
• Regarding Comparators
  a. Which were the most frequently assessed comparators (no exposure or lower exposure)?
  b. How were exposure levels assessed in comparators?
  c. Have possible co-exposures been considered?
• Regarding Outcome(s) and Data Analysis
  a. Are there more data on genetic or on epigenetic alterations?
  b. Which were the most frequently assessed genetic and epigenetic endpoints?
  c. Which techniques/assays were more commonly used to assess genetic and epigenetic endpoints?
  d. Which matrices were most frequently used for outcome assessment?
  e. If any, which other outcomes were analyzed simultaneously?
  f. Have possible confounding factors been considered?
  g. Which were the most frequent confounding factors considered?
  h. Which were the most frequent statistical approaches to data?
• Regarding Study Characteristics
  a. What type of studies have been developed (study design)?
  b. Which type of funding sources were the most common for these studies (e.g., public, private)?

## Methods

The present protocol has been prepared following the Collaboration for Environmental Evidence (CEE) Guidelines and Standards for Evidence Synthesis in Environmental Management [56] and the Reporting standards for systematic evidence synthesis in environmental research (ROSES) [57].

### Information Sources

To identify relevant peer-reviewed literature, a comprehensive search with no filters will be conducted in PubMed^1^ and Web of Science Core Collection^7^ electronic databases. The first database mentioned comprises biomedical journals and books, while the latter holds a multidisciplinary collection of indexed journals, books and conference proceedings. The combination of both will likely result in adequate coverage and un-biased sample of literature on the topic.

Further, grey literature will be also examined via Google,^8^ BASE^9^ and ProQuest,^10^ to include any existing technical reports, scientific opinions, position statements, white papers, thesis, conference papers, abstracts, and news on the topic. This search will ensure that documents that do not go through the scientific peer-review screening are also included in the SEM. The broad nature of grey literature searches (particularly in Google search engine) may potentially provide irrelevant results, but we are confident that this is a necessary step to not miss relevant information from non-traditional sources and ensure a comprehensive search.

### Evidence Search

Benchmarking: A benchmark list of relevant indexed publications was created to test the search strategy and assess its ability to retrieve relevant indexed publications from databases - serving to evaluate the comprehensiveness. Comprehensiveness was calculated as the percentage of benchmark publications retrieved by the search string: (number of benchmark publications retrieved/total number of publications in the benchmark list) × 100. For the elaboration of the benchmark list, authors have first identified reviews on the topic of the SEM independently and identified additional relevant papers through their reference lists. The final benchmark list, presented in Supplementary Table S1, included 75 publications: 13 reviews, 61 original research papers; and 1 additional manuscript identified in the screening of outputs of finished and ongoing international projects looking at the effects of early life exposures; namely, EXPOsOMICs, HELIX, ELEMENT, ELEAT, LIFEPATH, ENVIROGENOMARKERS, COPHES, DEMOCOPHES, PHIME, ENRIECO, DEER and HEALS.

Search string: Different keywords that can be used to describe the population (pregnant women and their offspring), exposure (tobacco use and/or ETS exposure), and outcomes (genetic and epigenetic alterations) were identified as presented in Supplementary Table S2.

A thorough analysis of the relevance of each keyword, also tested as wildcards when applicable, was carried out by examining the results obtained using each term alone, or combined (e.g., child vs. child AND child*), in a PubMed search. PECO-related keywords found to be more relevant, i.e., that returned the highest number of results, are indicated in bold in Supplementary Table S2. Then, to develop a reproducible and comprehensive search strategy, six variations of search strings combining the different keywords for population, exposure, and outcomes were tested for comprehensiveness and feasibility, as fully detailed in Supplementary Table S3. Building on authors’ previous knowledge that tobacco exposure is often analyzed as a confounder, all terms were sought in full-text. Search string SS6 - (tobacco OR smok* OR smoke OR cigarette) AND (pregnan* OR pregnancy OR pregnant OR gestation OR “*in utero*” OR intrauterine OR prenatal OR pre-natal OR perinatal OR antenatal OR ((maternal OR mother*) AND (newborn OR offspring OR child* OR fetus OR foetus OR fetal OR infant*))) AND (genetic OR genetic* OR epigenetic OR epigenetic* OR genotoxic OR genotoxic* OR cytogenetic* OR “DNA damage” OR “DNA methylation” OR “histone modification*” OR miRNA OR microRNA)) - was selected based on a balance of comprehensiveness and feasibility, as it retrieved 3756 articles in PubMed, with a comprehensiveness of 94.7%. This search string was tested in a second database - Web of Science (WoS) to assess cross-platform performance, and returned 3127 results, with a comprehensiveness of 80.3% (data not shown). Variability in comprehensiveness between PubMed and WoS reflects differences between the two databases, such as indexing practices, thesaurus use, and absence of a controlled vocabulary like MeSH in the latter [58, 59].

The grey literature search strategy was adapted to the requirements of each search engine. The following string “(pregnancy OR prenatal) AND (smoke OR tobacco) AND (genetic OR epigenetic),” composed of general search terms, will be used across Google, BASE, and ProQuest. In Google, the first 200 hits (non-sponsored) will be screened. In BASE and ProQuest, results will be filtered by document type (e.g., thesis, reports, conference abstracts, etc.), and the first 50 results of each document type will be reviewed. These thresholds were established to ensure transparency and reproducibility. A detailed description of the grey literature search strategy is provided in Supplementary Table S4.

No restrictions will be applied in these searches. If a search update is deemed necessary (if SEM writing takes over 18 months), the search in all databases will be repeated, filtered from the data of the last search.

### Records Management

Results obtained after bibliographic databases search will be imported to Endnote (Clarivate Analytics) and screened for duplicates, which will be removed. Results from grey literature search will be added to a dedicated records list, in Excel, and checked for duplicates considering DOI and/or website URL. It will also be checked if these records were not already found in PubMed and Web of Science.

Finally, reference details of all publications (bibliographic databases and grey literature) remaining after duplicate removal will be combined for further screening and receive a unique identification number that will be maintained throughout the SEM. The entire search process will be documented in a purposedly designed Excel spreadsheet by recording the name of the database searched, the date of the search, and publications obtained. Studies that arise from other sources, such as reference lists of included literature or reviews will be identified and recorded as “other sources”.

### Study Eligibility Criteria

Study eligibility criteria used to determine whether potential records shall be included or excluded in this SEM, based on the PECO statement, are presented in Table 2, and further detailed below.

• Population

**TABLE 2:** Inclusion and exclusion criteria to apply at screening stage (Worldwide, 2023).

Criterion	Inclusion	Exclusion
Population	Human	C1. *In vitro* study C2. *In vivo* study C3. Observational non-human (e.g., pets)
Exposure	Tobacco cigarettes Active (use) and passive (ETS exposure) *In utero* (including the periconceptional period)	C4. Exposure outside periconceptional and prenatal period (e.g., previous generations, after birth exposures) C5. Paternal exposure only (no data on maternal exposure) C6. Smoke exposure was not considered C7. Exposure to other tobacco products (electronic cigarettes, pipe, cigars or any product, other than cigarettes) or tobacco components alone (e.g., nicotine) C8. Data on smoke exposure is not presented
Comparator	Populations exposed to lower levels than the exposed population, or no exposure to tobacco	C9. No exposure comparator
		
Outcome	Alterations in the DNA structure and sequence Epigenetic changes (i.e., DNA methylation, histone modifications, and alterations of miRNA profiling)	C10. Outcomes other than genetic and epigenetic alterations of interest
Study design and language	Publications written in English language Studies containing specifically primary research data	C11. Non-English language C12. Case study (case report, case series) C13. Non-original research (e.g., reviews, commentary/letter to editor, editorial, study protocol)
Other		C14. Any other (the reason for exclusion will be described)

Only literature evaluating human populations will be included. No country restrictions were defined in the scope of this SEM. Studies conducted on the *in vitro* effects of tobacco, as well as on other animals or organisms (e.g., rats, cats, dogs, rats) will be excluded.

• Exposure

Both tobacco use and/or ETS exposure *in utero* will be considered. Other source(s)/product(s) apart from cigarettes (e.g., electronic cigarettes, cigars, etc.) will not be considered.

• Comparator(s)

Comparators will include population not exposed (*in utero*) or exposed to lower levels of tobacco and/or ETS than the exposed population.

• Outcome(s)

The following outcomes related to the included populations will be considered: alterations in the DNA structure and sequence, and epigenetic changes such as DNA methylation, histone modifications, and alterations of miRNA profiling. There will be no restrictions on analytical methods.

• Study design and language

Studies containing specifically primary research data investigating the link between tobacco exposure and genetic/epigenetic alterations *in utero* will be considered for inclusion; case studies (e.g., case report, case series) and non-original research (e.g., reviews, commentary/letter to editor, editorial, study protocols) will be sorted for exclusion. Publications written in language apart from English will be excluded from this systematic evidence map due to limited resources–even though this is certainly a limitation as it may exclude relevant data from low and middle income countries, previous research [60] suggests that such exclusion of non-English publications may have a minimal impact on the SEM outcomes.

### Study Selection

**FIGURE 1:**
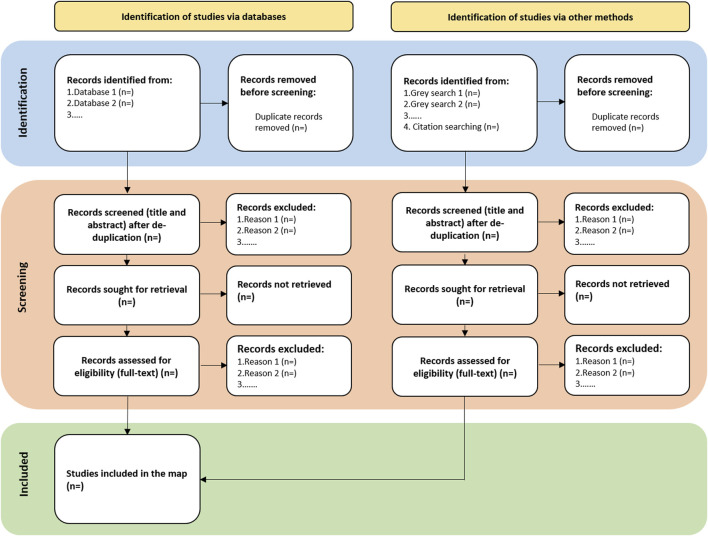
Preferred Reporting Items for Systematic reviews and Meta-Analyses (PRISMA) flow diagram (adapted from Page et al. [61]; Worldwide, 2023).

The title and abstract of all records will be initially screened for compliance with inclusion/exclusion criteria. At this stage, to minimize the risk of erroneous exclusion due to incomplete or insufficiently detailed abstracts, any records that are unclear or lack key information—such as details on methodology, study population, or relevant outcomes—will be retained for full-text screening. Full-texts of those found eligible will be retrieved and reviewed against the previously detailed inclusion/exclusion criteria. At both stages, two independent reviewers will perform the screening. Discrepant screening results will be resolved by discussion with a third reviewer. If some full-text cannot be obtained, it will be solicited to the corresponding author by e-mail; if no answer is obtained within 2 weeks, the study will be included using the information available in the abstract. The number of studies retrieved through our search, as well as the number of evaluated, included and excluded studies at each stage of screening, will be documented in a study flow diagram (Figure 1). The reasons for exclusion of studies, at title and abstract or full-text analysis will be recorded to a standardized codification (Table 2).

Reference mining of included full-texts will be checked for possible inclusion on SEM.

### Data Extraction and Coding

Data will be extracted from included full-text studies using a form (Excel) developed by the authors. Information to be collected is presented in Table 3. This form will ensure that all pertinent data needed to answer the above-mentioned queries (presented in the SEM objectives’ section) will be collected in a consistent way, including bibliographic information, study characteristics, and PECO components. Data will be provided in raw format (as indicated in the publication) and in pre-specified controlled (standardized) vocabulary/terms in separate tables. A full list of used terms and their definitions will be provided in a glossary (codebook). The use of controlled vocabulary will enable search based on matches to specific terms across the entire dataset, avoid ambiguous data annotation, control for data heterogeneity and increase consistency [62].

**TABLE 3:** Key data extraction items (Worldwide, 2023).

General Information	Study record number (assigned by the reviewers) Publication URL or DOI Corresponding author e-mail address Year of publication Publication type (e.g., peer-reviewed research paper, pre-print research paper, thesis, conference paper, conference abstract, book chapter, report, book)
Funding	Funding source (i.e., public funding, non-profit foundations, private corporations) Conflict of interest (i.e., yes, no, not reported)
Population	Date of the study - sampling time frame (from mm.yyyy to mm.yyyy) Location (i.e., city, country) Population life stage (i.e., newborns, children, adolescents, adults, elder) Sample size (number of enrolled individuals)
Study design	Study design (i.e., cross-sectional, prospective cohort, retrospective cohort, other)
Exposure	Is tobacco exposure the main topic, or studied as a confounder? (i.e., main, confounder (to which exposure)) Type of tobacco exposure (i.e., active smoking, ETS exposure) Tobacco exposure assessment (i.e., questionnaire, biomarker, modelling) IF questionnaire (i.e., national, international; validated, non-validated, no info on validation) IF biomarker (matrix; endpoint; assay) Timing of tobacco exposure (i.e., combinations of: periconception, entire pregnancy, 1st, 2nd and 3rd trimesters)
Comparator	Level of exposure (i.e*.,* no exposure, lower exposure, before-after comparison) Exposure assessment (i.e., same as exposed, other (which))
Outcome	Global outcome (i.e., genetics, epigenetics, both) Endpoint (e.g., DNA strand breaks, FPG-sensitive sites, micronuclei, sister chromatid exchange, global DNA methylation) Endpoint matrix Endpoint assessment technique (e.g., comet assay, PFG-modified comet assay, microscopy, flow cytometry) Other simultaneous endpoints – different of those of interest (e.g., asthma diagnosis, neurodevelopment alterations)
Confounding and Statistics (if tobacco is the main exposure studied)	Confounding consideration (i.e.*,* no, yes (which: e.g., age, sex, diet; when: i.e., study design, statistical analysis, both)) Co-exposures consideration (i.e., no, yes (which)) Statistical tests – association of exposure with endpoint (e.g., t-test, Mann-Whitney, linear regression)

Data extraction and coding will be conducted by one reviewer, with a second reviewer confirming the accuracy and completeness of extracted and coded data. Particular attention will be given to identify possible multiple reports from a single study (e.g., several publications, conference abstracts); in these cases, to improve database readability, and avoid misinterpretation of duplicated entries, the database will clearly identify related rows.

### Data Querying, Visualization and Synthesis of Results

The use of filter table columns, sort/order, and search functions of Excel will warrant end-users to easily identify and find specific study details in a queryable user-friendly database (single table in an Excel spreadsheet) referenced to primary studies; a hyperlink to the reference website will also be included to facilitate publication tracking. To avoid loss of data and referential integrity [62], cells will house single data, and multiple outcomes/populations in the same study will be presented in different rows. The full database, controlled vocabulary definitions, and instructions on how to interact with the database will be made available as Supplementary Material.

Data will be analyzed using descriptive statistics and summarized in tables, bubble graphs, heat maps or other type of diagram to support the narrative synthesis and summarize the evidence landscape on the short and long-term genetic and epigenetic effects in the offspring resulting from prenatal human exposure to tobacco (tobacco use and/or ETS exposure).

### Pilot Study

To test the feasibility of the protocol described above, a pilot study has been carried out focusing on publications published in 2020. This approach was used to efficiently evaluate and refine key components of the workflow, including the application of inclusion and exclusion criteria, the structure and usability of data extraction templates, and the clarity of categorization schemes. By working with a manageable dataset limited to a single publication year, the research team was able to identify unanticipated issues early in the process. Although the one-year scope may not fully capture temporal variation in study design or terminology, and some refinements may be needed as the evidence mapping extends to a broader timeframe, any such adjustments will be transparently documented. Nonetheless, the insights gained from the pilot have made a substantial contribution to strengthening the protocol’s methodological foundation. Details on results obtained and fine-tuning of different stages of the protocol are presented below.

## Results

As depicted in Supplementary Figure S1, following the above-described search strategy, 192 papers were retrieved from PubMed and 189 papers from Web of Science, all published in 2020. Grey databases recovered 229 publications (48 in BASE, 21 in ProQuest and 160 in Google), that included peer-reviewed papers, thesis, books and reports.

Duplicate screening was performed in the first stage in Endnote (PubMed and Web of Science results) and in Excel in a second stage (combination with grey literature). After duplicate removal, 329 titles and abstracts were screened, and 75 progressed to full-text analysis; out of these, 7 met all the established eligibility criteria, and therefore, were used to test the extraction datasheets.

At the screening stage, it became evident that the initially defined eligibility criteria (not shown) were insufficient to guide consistent and transparent decision-making. As a result, authors developed a detailed and comprehensive exclusion list, as presented in Table 2. The refined framework enabled authors to objectively apply exclusion criteria across both stages of the screening process.

Further adjustments were also required to ensure the accuracy and consistency of data extraction. More specifically, it was found necessary to prepare a guidance note document to support reviewers in the correct application of controlled vocabulary, especially for terms related to study design and analytical methodologies used in outcome assessment. This document provided standardized definitions and decision rules, helping to minimize ambiguity and inter-reviewer variability, thereby contributing to greater reliability in coding and categorization during the extraction process.

## Conclusion

The establishment of a protocol to perform the planned SEM ensures, *a priori*, that the proposed workflow and methodology will be conducted in a transparent, impartial manner, in line with the CEE Guidelines, Standards for Evidence Synthesis in Environmental Management, and the ROSES reporting standards. The pilot study here presented was particularly valuable in illustrating procedures, clarifying doubts, settling criteria, and identifying possible constraints and strategies to overcome them.

Upon completion, this SEM will provide a structured and comprehensive overview of the existing evidence on the short and long-term genetic and epigenetic outcomes in offspring following *in utero* exposure to tobacco and/or ETS.

Unlike traditional systematic reviews, which typically narrow their focus to evaluate specific outcomes or exposures, this SEM will map the full breadth of research in this topic, including exposure type, study design, biomarkers assessed, outcomes, and populations studied. This will allow the identification of under-researched areas, data gaps (e.g., evidence available and effects in low- and middle-income countries, underrepresented biomarkers, long-term follow-ups; potential equity concerns; needs for targeted research efforts) and clusters of data rich evidence suitable for future in-depth reviews.

While this SEM does not assess the quality of individual studies, ensuring that the existing scientific literature, regardless of quality, is not overlooked or underutilized, descriptive data such as study design, sample size, and exposure/outcome measures will be reported to support transparency and help readers appraise the evidence quality informally. The organization of evidence in a searchable, queryable format will serve as a resource for environmental health researchers, geneticists, and health practitioners, particularly those involved in obstetric, neonatal, and pediatric care, as well as policymakers responsible for public health interventions on tobacco control and pregnancy-related health outcomes. This SEM will help prioritize areas where implementation of smoking cessation guidelines could have the greatest impact.

## Footnotes

1
https://pubmed.ncbi.nlm.nih.gov

2
https://www.ebsco.com/products/research-databases

3
https://www.epistemonikos.org

4
https://www.crd.york.ac.uk/prospero/

5
https://www.cos.io/products/osf-registries

6
https://zenodo.org

7
https://www.webofscience.com/wos/woscc/basic-search

8
https://www.google.com

9
https://www.base-search.net/

10
https://www.proquest.com/
