# Supplementary material for: Genetic and Epigenetic Alterations Associated With Human Prenatal Tobacco and Environmental Tobacco Smoke Exposure: Protocol for a Systematic Evidence Map
Source: Public Health Rev. 2025 Jul 14;46:1606742. doi: 10.3389/phrs.2025.1606742 (PMC12301260; doi:10.3389/phrs.2025.1606742)
Supplement: Supplementary file 1 [file DataSheet1.docx]

| **Table S1.** Benchmark list used to assess comprehensiveness | | |
| --- | --- | --- |
| 1 | M.M. Ammenheuser, A.B. Berenson, N.J. Stiglich, E.B.W. Jr., J.B.W. Jr. Elevated frequencies of hprt mutant lymphocytes in cigarette-smoking mothers and their newborns. Mutat Res-Fund Mol M, 304 (2) (1994), pp.285-94, [https://doi.org/10.1016/0027-5107(94)90221-6](http://www.google.com) | O |
| 2 | G. Ardito, L. Lamberti, E. Ansaldi, P. Ponzetto. Sister-chromatid exchanges in cigarette-smoking human females and their newborns. Mutat Res, 78 (2) (1980), pp. 209-212, [https://doi.org/10.1016/0165-1218(80)90100-7](https://doi.org/10.1289/ehp.10144) | O |
| 3 | J.P. Arnould, P. Verhoest, V. Bach, J.P. Libert, J. Belegaud. Detection of benzo[a]pyrene-DNA adducts in human placenta and umbilical cord blood. Hum Exp Toxicol, 16 (12) (1997), pp. 716-721, <https://doi.org/10.1177/096032719701601204> | O |
| 4 | Y. Ba, H. Yu, F. Liu, X. Geng, C. Zhu, Q. Zhu, et al. Relationship of folate, vitamin B12 and methylation of insulin-like growth factor-II in maternal and cord blood. Eur J Clin Nutr, 65 (4) (2011), pp. 480-485, [https://doi.org/10.1038/ejcn.2010.294](https://doi.org/10.1016/j.scitotenv.2007.06.003) | O |
| 5 | S. Barua, M.A. Junaid. Lifestyle, pregnancy and epigenetic effects. 7 (1) (2015), pp. 85-102, [https://doi.org/10.2217/epi.14.71](https://doi.org/10.1016/0027-5107(94)90221-6) | R |
| 6 | L.M. Bennett, Y. Wang, M.J. Ramsey, G.F. Harger, W.L. Bigbee, J.D. Tucker. Cigarette smoking during pregnancy: Chromosome translocations and phenotypic susceptibility in mothers and newborns. Mutat Res - Gen Tox, 696 (1) (2010), pp. 81-88, [https://doi.org/10.1016/j.mrgentox.2009.12.015](https://doi.org/10.4161/15592294.2014.971593) | O |
| 7 | W.L. Bigbee, R.D. Day, S.G. Grant, P. Keohavong, L. Xi, L. Zhang, et al. Impact of maternal lifestyle factors on newborn HPRT mutant frequencies and molecular spectrum — Initial results from the Prenatal Exposures and Preeclampsia Prevention (PEPP) Study. Mutat Res-Fund Mol M, 431 (2) (1999), pp. 279-289, [https://doi.org/10.1016/S0027-5107(99)00172-4](http://www.proquest.com) | O |
| 8 | C.V. Breton, H.M. Byun, M. Wenten, F. Pan, A. Yang, F.D. Gilliland. Prenatal tobacco smoke exposure affects global and gene-specific DNA methylation. Am J Respir Crit Care Med, 180 (5) (2009), pp. 462-467, <https://doi.org/10.1164/rccm.200901-0135OC> | O |
| 9 | C.V. Breton, M.T. Salam, F.D. Gilliland. Heritability and role for the environment in DNA methylation in AXL receptor tyrosine kinase. Epigenetics, 6 (7) (2011), pp. 895-898, [https://doi.org/10.4161/epi.6.7.15768](https://doi.org/10.1038/ejcn.2010.294) | O |
| 10 | C.V. Breton, K.D. Siegmund, B.R. Joubert, X. Wang, W. Qui, V. Carey, et al. Prenatal tobacco smoke exposure is associated with childhood DNA CpG methylation. PLOS ONE, 9 (6) (2014), pp. e99716, [https://doi.org/10.1371/journal.pone.0099716](https://doi.org/10.2217/epi.14.71) | O |
| 11 | D. Chhabra, S. Sharma, A.T. Kho, R. Gaedigk, C.A. Vyhlidal, J.S. Leeder, et al. Fetal lung and placental methylation is associated with in utero nicotine exposure. Epigenetics, 9 (11) (2014), pp. 1473-1484, [https://doi.org/10.4161/15592294.2014.971593](https://www.base-search.net/) | O |
| 12 | R.A. de la Chica, I. Ribas, J. Giraldo, J. Egozcue, C. Fuster. Chromosomal instability in amniocytes from fetuses of mothers who smoke. JAMA, 293 (10) (2005), pp. 1212-1222, [https://doi.org/10.1001/jama.293.10.1212](https://doi.org/10.1093/mutage/13.1.99) | O |
| 13 | D.M. Demarini, R.J. Preston. Smoking while pregnant: transplacental mutagenesis of the fetus by tobacco smoke. JAMA, 293 (10) (2005), pp. 1264-1265, <https://doi.org/10.1001/jama.293.10.1264> | R |
| 14 | R.B. Everson. A review of approaches to the detection of genetic damage in the human fetus. Environ Health Perspect, 74 (1987), pp. 109-117, [https://doi.org/10.1289/ehp.8774109](https://doi.org/10.1016/j.mrgentox.2009.12.015) | R |
| 15 | R.B. Everson, E. Randerath, R.M. Santella, R.C. Cefalo, T.A. Avitts, K. Randerath. Detection of smoking-related covalent DNA adducts in human placenta. Science, 231 (4733) (1986), pp. 54, [https://doi.org/10.1126/science.3941892](https://doi.org/10.1016/S0027-5107(99)00172-4) | O |
| 16 | S. Fa, T.V. Larsen, K. Bilde, T.F. Daugaard, E.H. Ernst, R.H. Olesen, et al. Assessment of global DNA methylation in the first trimester fetal tissues exposed to maternal cigarette smoking. Clin Epigenetics, 8 (1) (2016), pp. 128, [https://doi.org/10.1186/s13148-016-0296-0](https://doi.org/10.1007/s40572-015-0045-0) | O |
| 17 | B.A. Finette, J.P. O'Neill, P.M. Vacek, R.J. Albertini. Gene mutations with characteristic deletions in cord blood T lymphocytes associated with passive maternal exposure to tobacco smoke. Nat Med, 4 (10) (1998), pp. 1144-1151, [https://doi.org/10.1038/2640](https://doi.org/10.4161/epi.6.7.15768) | O |
| 18 | B.A. Finette, T. Poseno, P.M. Vacek, R.J. Albertini. The effects of maternal cigarette smoke exposure on somatic mutant frequencies at the hprt locus in healthy newborns. Mutat Res-Fund Mol M, 377 (1) (1997), pp. 115-123, <https://doi.org/10.1016/S0027-5107(97)00069-9> | O |
| 19 | J.D. Flom, J.S. Ferris, Y. Liao, P. Tehranifar, C.B. Richards, Y.H. Cho, et al. Prenatal smoke exposure and genomic DNA methylation in a multiethnic birth cohort. Cancer Epidemiol Biomarkers Prev, 20 (12) (2011), pp. 2518-2523, [https://doi.org/10.1158/1055-9965.Epi-11-0553](https://doi.org/10.1289/ehp.8774109) | O |
| 20 | K. Gibbs, J.M. Collaco, S.A. McGrath-Morrow. Impact of Tobacco Smoke and Nicotine Exposure on Lung Development. Chest, 149 (2) (2016), pp. 552-561, [https://doi.org/10.1378/chest.15-1858](https://doi.org/10.1126/science.3941892) | R |
| 21 | B.B. Green, C.J. Marsit. Select Prenatal Environmental Exposures and Subsequent Alterations of Gene-Specific and Repetitive Element DNA Methylation in Fetal Tissues. Curr Environ Health Rep, 2 (2) (2015), pp. 126-136, [https://doi.org/10.1007/s40572-015-0045-0](https://doi.org/10.1093/molehr/gas050) | R |
| 22 | R. Guerrero-Preston, L.R. Goldman, P. Brebi-Mieville, C. Ili-Gangas, C. Lebron, F.R. Witter, et al. Global DNA hypomethylation is associated with in utero exposure to cotinine and perfluorinated alkyl compounds. Epigenetics, 5 (6) (2010), pp. 539-546, [https://doi.org/10.4161/epi.5.6.12378](https://doi.org/10.1158/1055-9965.Epi-11-0553) | O |
| 23 | G. Herberth, M. Bauer, M. Gasch, D. Hinz, S. Röder, S. Olek, et al. Maternal and cord blood miR-223 expression associates with prenatal tobacco smoke exposure and low regulatory T-cell numbers. J Allergy Clin Immunol, 133 (2) (2014), pp. 543-550, [https://doi.org/10.1016/j.jaci.2013.06.036](https://doi.org/10.1016/0165-1218(80)90100-7) | O |
| 24 | K. Husgafvel-Pursiainen. Genotoxicity of environmental tobacco smoke: a review. Mutat Res, 567 (2-3) (2004), pp. 427-445, <https://doi.org/10.1016/j.mrrev.2004.06.004> | R |
| 25 | B.R. Joubert, J.F. Felix, P. Yousefi, K.M. Bakulski, A.C. Just, C. Breton, et al. DNA Methylation in Newborns and Maternal Smoking in Pregnancy: Genome-wide Consortium Meta-analysis. Am J Hum Genet, 98 (4) (2016), pp. 680-696, [https://doi.org/10.1016/j.ajhg.2016.02.019](https://doi.org/10.1038/2640) | O |
| 26 | B.R. Joubert, S.E. Håberg, D.A. Bell, R.M. Nilsen, S.E. Vollset, O. Midttun, et al. Maternal smoking and DNA methylation in newborns: in utero effect or epigenetic inheritance? Cancer Epidemiology, Biomarkers & Prevention, 23 (6) (2014), pp. 1007-1017, [https://doi.org/10.1158/1055-9965.Epi-13-1256](https://doi.org/10.1096/fj.11-201194) | O |
| 27 | B.R. Joubert, S.E. Håberg, R.M. Nilsen, X. Wang, S.E. Vollset, S.K. Murphy, et al. 450K epigenome-wide scan identifies differential DNA methylation in newborns related to maternal smoking during pregnancy. Environ Health Perspect, 120 (10) (2012), pp. 1425-1431, [https://doi.org/10.1289/ehp.1205412](https://doi.org/10.4161/epi.5.6.12378) | O |
| 28 | G. Kaur, R. Begum, S. Thota, S. Batra. A systematic review of smoking-related epigenetic alterations. Arch Toxicol, 93 (10) (2019), pp. 2715-2740, [https://doi.org/10.1007/s00204-019-02562-y](https://doi.org/10.1378/chest.15-1858) | R |
| 29 | P. Keohavong, L. Xi, R.D. Day, L. Zhang, S.G. Grant, B.W. Day, et al. HPRT gene alterations in umbilical cord blood T-lymphocytes in newborns of mothers exposed to tobacco smoke during pregnancy. Mutat Res, 572 (1-2) (2005), pp. 156-166, <https://doi.org/10.1016/j.mrfmmm.2005.01.014> | O |
| 30 | V.S. Knopik, M.A. Maccani, S. Francazio, J.E. McGeary. The epigenetics of maternal cigarette smoking during pregnancy and effects on child development. Dev Psychopathol, 24 (4) (2012), pp. 1377-1390, [https://doi.org/10.1017/s0954579412000776](https://doi.org/10.1016/j.ajhg.2016.02.019) | R |
| 31 | J. Laubenthal, O. Zlobinskaya, K. Poterlowicz, A. Baumgartner, M.R. Gdula, E. Fthenou, et al. Cigarette smoke-induced transgenerational alterations in genome stability in cord blood of human F1 offspring. FASEB J, 26 (10) (2012), pp. 3946-3956, [https://doi.org/10.1096/fj.11-201194](https://doi.org/10.1371/journal.pone.0099716) | O |
| 32 | K.W. Lee, R. Richmond, P. Hu, L. French, J. Shin, C. Bourdon, et al. Prenatal exposure to maternal cigarette smoking and DNA methylation: epigenome-wide association in a discovery sample of adolescents and replication in an independent cohort at birth through 17 years of age. Environ Health Perspect, 123 (2) (2015), pp. 193-199, [https://doi.org/10.1289/ehp.1408614](https://doi.org/10.1186/s13148-016-0296-0) | O |
| 33 | M.S. Lundberg, G.K. Livingston. Sister-chromatid exchange frequency in lymphocytes of smoking and nonsmoking mothers and their newborn infants. Mutat Res Lett, 121 (3) (1983), pp. 241-246, [https://doi.org/10.1016/0165-7992(83)90209-9](https://doi.org/10.1001/jama.293.10.1212) | O |
| 34 | K. Lundgren, J.M. Lambert, D. Schreinemachers, R.B. Everson. Effects of 5-bromo-2-deoxyuridine concentration and alpha-naphthoflavone on the association between smoking and the frequency of sister-chromatid exchanges in lymphocytes from maternal and cord blood. Mutat Res, 188 (3) (1987), pp. 223-231, <https://doi.org/10.1016/0165-1218(87)90093-0> | O |
| 35 | J.Z. Maccani, D.C. Koestler, E.A. Houseman, C.J. Marsit, K.T. Kelsey. Placental DNA methylation alterations associated with maternal tobacco smoking at the RUNX3 gene are also associated with gestational age. Epigenomics, 5 (6) (2013), pp. 619-630, [https://doi.org/10.2217/epi.13.63](https://doi.org/10.1289/ehp.1205412) | O |
| 36 | M.A. Maccani, M. Avissar-Whiting, C.E. Banister, B. McGonnigal, J.F. Padbury, C.J. Marsit. Maternal cigarette smoking during pregnancy is associated with downregulation of miR-16, miR-21, and miR-146a in the placenta. Epigenetics, 5 (7) (2010), pp. 583-589, [https://doi.org/10.4161/epi.5.7.12762](https://doi.org/10.1016/j.jaci.2013.06.036) | O |
| 37 | M.A. Maccani, V.S. Knopik. Cigarette smoke exposure-associated alterations to non-coding RNA. Frontiers in Genetics, 3 (2012), pp. 53, [https://doi.org/10.3389/fgene.2012.00053](https://doi.org/10.1289/ehp.1408614) | O |
| 38 | M.A. Maccani, C.J. Marsit. Exposure and fetal growth-associated miRNA alterations in the human placenta. Clin Epigenetics, 2 (2) (2011), pp. 401-404, [https://doi.org/10.1007/s13148-011-0046-2](https://doi.org/10.1017/s0954579412000776) | O |
| 39 | D.K. Manchester, J.A. Nicklas, J.P. O'Neill, M.J. Lippert, S.G. Grant, R.G. Langlois, et al. Sensitivity of somatic mutations in human umbilical cord blood to maternal environments. Environ Mol Mutagen, 26 (3) (1995), pp. 203-212, <https://doi.org/10.1002/em.2850260304> | O |
| 40 | C.A. Markunas, Z. Xu, S. Harlid, P.A. Wade, R.T. Lie, J.A. Taylor, et al. Identification of DNA methylation changes in newborns related to maternal smoking during pregnancy. Environ Health Perspect, 122 (10) (2014), pp. 1147-1153, [https://doi.org/10.1289/ehp.1307892](https://doi.org/10.2217/epi.13.63) | O |
| 41 | K.B. Michels, H.R. Harris, L. Barault. Birthweight, Maternal Weight Trajectories and Global DNA Methylation of LINE-1 Repetitive Elements. PLOS ONE, 6 (9) (2011), pp. e25254, [https://doi.org/10.1371/journal.pone.0025254](https://doi.org/10.1007/s00204-019-02562-y) | O |
| 42 | E. Morales, N. Vilahur, L.A. Salas, V. Motta, M.F. Fernandez, M. Murcia, et al. Genome-wide DNA methylation study in human placenta identifies novel loci associated with maternal smoking during pregnancy. Int J Epidemiol, 45 (5) (2016), pp. 1644-1655, [https://doi.org/10.1093/ije/dyw196](https://doi.org/10.1158/1055-9965.Epi-13-1256) | O |
| 43 | S.K. Murphy, A. Adigun, Z. Huang, F. Overcash, F. Wang, R.L. Jirtle, et al. Gender-specific methylation differences in relation to prenatal exposure to cigarette smoke. Gene, 494 (1) (2012), pp. 36-43, [https://doi.org/10.1016/j.gene.2011.11.062](https://doi.org/10.1111/cea.12108) | O |
| 44 | M. Neri, D. Ugolini, S. Bonassi, A. Fucic, N. Holland, L.E. Knudsen, et al. Children's exposure to environmental pollutants and biomarkers of genetic damage. II. Results of a comprehensive literature search and meta-analysis. Mutat Res, 612 (1) (2006), pp. 14-39, [https://doi.org/10.1016/j.mrrev.2005.04.003](https://doi.org/10.4161/epi.5.7.12762) | R |
| 45 | C.H. Nielsen, A. Larsen, A.L. Nielsen. DNA methylation alterations in response to prenatal exposure of maternal cigarette smoking: A persistent epigenetic impact on health from maternal lifestyle? Arch Toxicol, 90 (2) (2016), pp. 231-245, <https://doi.org/10.1007/s00204-014-1426-0> | R |
| 46 | B. Novakovic, J. Ryan, N. Pereira, B. Boughton, J.M. Craig, R. Saffery. Postnatal stability, tissue, and time specific effects of AHRR methylation change in response to maternal smoking in pregnancy. Epigenetics, 9 (3) (2014), pp. 377-386, [https://doi.org/10.4161/epi.27248](https://doi.org/10.1016/0165-7992(83)90209-9) | O |
| 47 | V.K. Patil, J.W. Holloway, H. Zhang, N. Soto-Ramirez, S. Ewart, S.H. Arshad, et al. Interaction of prenatal maternal smoking, interleukin 13 genetic variants and DNA methylation influencing airflow and airway reactivity. Clin Epigenetics, 5 (1) (2013), pp. 22-22, [https://doi.org/10.1186/1868-7083-5-22](https://doi.org/10.1093/ije/dyw196) | O |
| 48 | F.P. Perera, W. Jedrychowski, V. Rauh, R.M. Whyatt. Molecular epidemiologic research on the effects of environmental pollutants on the fetus. Environ Health Perspect, 107 (Suppl 3) (1999), pp. 451-460, [https://doi.org/10.1289/ehp.99107s3451](https://doi.org/10.1289/ehp.1307892) | O |
| 49 | F.P. Perera, D. Tang, V. Rauh, Y.H. Tu, W.Y. Tsai, M. Becker, et al. Relationship between polycyclic aromatic hydrocarbon-DNA adducts, environmental tobacco smoke, and child development in the World Trade Center cohort. Environ Health Perspect, 115 (10) (2007), pp. 1497-1502, [https://doi.org/10.1289/ehp.10144](https://doi.org/10.1371/journal.pone.0025254) | O |
| 50 | F.P. Perera, D. Tang, Y.-H. Tu, L.A. Cruz, M. Borjas, T. Bernert, et al. Biomarkers in maternal and newborn blood indicate heightened fetal susceptibility to procarcinogenic DNA damage. Environ Health Perspect, 112 (10) (2004), pp. 1133-1136, <https://doi.org/10.1289/ehp.6833> | O |
| 51 | F. Pirini, E. Guida, F. Lawson, A. Mancinelli, R. Guerrero-Preston. Nuclear and mitochondrial DNA alterations in newborns with prenatal exposure to cigarette smoke. Int J Environ Res Public Health, 12 (2) (2015), pp. 1135-1155, [https://doi.org/10.3390/ijerph120201135](https://doi.org/10.1007/s13148-011-0046-2) | R |
| 52 | J.M. Pluth, M.J. Ramsey, J.D. Tucker. Role of maternal exposures and newborn genotypes on newborn chromosome aberration frequencies. Mutat Res - Genet Toxicol Environ Mutagen, 465 (1) (2000), pp. 101-111, [https://doi.org/10.1016/S1383-5718(99)00217-X](https://doi.org/10.1016/j.mrrev.2005.04.003) | O |
| 53 | R.C. Richmond, A.J. Simpkin, G. Woodward, T.R. Gaunt, O. Lyttleton, W.L. McArdle, et al. Prenatal exposure to maternal smoking and offspring DNA methylation across the lifecourse: findings from the Avon Longitudinal Study of Parents and Children (ALSPAC). Hum Mol Genet, 24 (8) (2015), pp. 2201-2217, [https://doi.org/10.1093/hmg/ddu739](https://doi.org/10.1016/0165-1218(89)90024-4) | O |
| 54 | R.C. Richmond, M. Suderman, R. Langdon, C.L. Relton, G. Davey Smith. DNA methylation as a marker for prenatal smoke exposure in adults. Int J Epidemiol, 47 (4) (2018), pp. 1120-1130, [https://doi.org/10.1093/ije/dyy091](https://doi.org/10.4161/epi.27248) | O |
| 55 | S. Şardaş, B. Karahalil, D. Akyol, S. Kükner, A.E. Karakaya. The effect of smoking on sister chromatid exchange rate of newborn infants born to smoking mothers. Mutat Res Genet Toxicol, 341 (4) (1995), pp. 249-253, <https://doi.org/10.1016/0165-1218(95)90096-9> | O |
| 56 | S. Şardaş, D. Walker, D. Akyol, A.E. Karakaya. Assessment of smoking-induced DNA damage in lymphocytes of smoking mothers of newborn infants using the alkaline single-cell gel electrophoresis technique. Mutat Res - Environ Mut Rel Sub, 335 (3) (1995), pp. 213-217, [https://doi.org/10.1016/0165-1161(95)00023-2](https://doi.org/10.1289/ehp.99107s3451) | O |
| 57 | R. Seshadri, E. Baker, G.R. Sutherland. Sister-chromatid exchange (SCE) analysis in mothers exposed to DNA-damaging agents and their newborn infants. Mutat Res-Envir Muta, 97 (2) (1982), pp. 139-146, [https://doi.org/10.1016/0165-1161(82)90011-5](https://doi.org/10.1016/S1383-5718(99)00217-X) | O |
| 58 | M. Sorsa, K. Husgafvel-Pursiainen. Assessment of passive and transplacental exposure to tobacco smoke. IARC Sci Publ, 89 (1988), pp. 129-132. | O |
| 59 | M. Sorsa, K. Husgafvel-Pursiainen, H. Järventaus, K. Koskimies, H. Salo, H. Vainio. Cytogenetic effects of tobacco smoke exposure among involuntary smokers. Mutat Res - Genet Toxicol, 222 (2) (1989), pp. 111-116, [https://doi.org/10.1016/0165-1218(89)90024-4](https://doi.org/10.3389/fgene.2012.00053) | O |
| 60 | R.J. Srám, K. Podrazilová, J. Dejmek, G. Mracková, T. Pilcík. Single cell gel electrophoresis assay: sensitivity of peripheral white blood cells in human population studies. Mutagenesis, 13 (1) (1998), pp. 99-103, [https://doi.org/10.1093/mutage/13.1.99](https://doi.org/10.3390/ijerph120201135) | O |
| 61 | M. Suter, A. Abramovici, K. Aagaard-Tillery. Genetic and epigenetic influences associated with intrauterine growth restriction due to in utero tobacco exposure. Pediatr Endocrinol Rev, 8 (2) (2010), pp. 94-102. | O |
| 62 | M. Suter, A. Abramovici, L. Showalter, M. Hu, C.D. Shope, M. Varner, et al. In utero tobacco exposure epigenetically modifies placental CYP1A1 expression. Metabolism, 59 (10) (2010), pp. 1481-1490, <https://doi.org/10.1016/j.metabol.2010.01.013> | O |
| 63 | M. Suter, J. Ma, A. Harris, L. Patterson, K.A. Brown, C. Shope, et al. Maternal tobacco use modestly alters correlated epigenome-wide placental DNA methylation and gene expression. 6 (11) (2011), pp. 1284-1294, [https://doi.org/10.4161/epi.6.11.17819](https://doi.org/10.1016/0165-1161(95)00023-2) | O |
| 64 | M.A. Suter, K. Aagaard. What changes in DNA methylation take place in individuals exposed to maternal smoking in utero? Epigenomics, 4 (2) (2012), pp. 115-118, [https://doi.org/10.2217/epi.12.7](https://doi.org/10.1093/ije/dyy091) | R |
| 65 | M.A. Suter, A.M. Anders, K.M. Aagaard. Maternal smoking as a model for environmental epigenetic changes affecting birthweight and fetal programming. Mol Hum Reprod, 19 (1) (2013), pp. 1-6, [https://doi.org/10.1093/molehr/gas050](https://doi.org/10.1186/s12916-020-01686-8) | R |
| 66 | P. Tehranifar, H.C. Wu, J.A. McDonald, F. Jasmine, R.M. Santella, I. Gurvich, et al. Maternal cigarette smoking during pregnancy and offspring DNA methylation in midlife. Epigenetics, 13 (2) (2018), pp. 129-134, [https://doi.org/10.1080/15592294.2017.1325065](https://doi.org/10.1016/j.gene.2011.11.062) | O |
| 67 | M.B. Terry, J.S. Ferris, R. Pilsner, J.D. Flom, P. Tehranifar, R.M. Santella, et al. Genomic DNA methylation among women in a multiethnic New York City birth cohort. Cancer Epidemiol Biomarkers Prev, 17 (9) (2008), pp. 2306-2310, [https://doi.org/10.1158/1055-9965.Epi-08-0312](https://doi.org/10.1016/0165-1161(82)90011-5) | O |
| 68 | E.W. Tobi, B.T. Heijmans, D. Kremer, H. Putter, H.A. Delemarre-van de Waal, M.J.J. Finken, et al. DNA methylation of IGF2, GNASAS, INSIGF and LEP and being born small for gestational age. Epigenetics, 6 (2) (2011), pp. 171-176, <https://doi.org/10.4161/epi.6.2.13516> | O |
| 69 | M. Toledo-Rodriguez, S. Lotfipour, G. Leonard, M. Perron, L. Richer, S. Veillette, et al. Maternal smoking during pregnancy is associated with epigenetic modifications of the brain-derived neurotrophic factor-6 exon in adolescent offspring. Am J Med Genet B Neuropsychiatr Genet, 153b (7) (2010), pp. 1350-1354, [https://doi.org/10.1002/ajmg.b.31109](https://doi.org/10.2217/epi.12.7) | O |
| 70 | M. Vives-Usano, C. Hernandez-Ferrer, L. Maitre, C. Ruiz-Arenas, S. Andrusaityte, E. Borràs, et al. In utero and childhood exposure to tobacco smoke and multi-layer molecular signatures in children. BMC Med, 18 (1) (2020), pp. 243, [https://doi.org/10.1186/s12916-020-01686-8](https://doi.org/10.1186/1868-7083-5-22) | OP Helix project |
| 71 | I.J. Wang, S.L. Chen, T.P. Lu, E.Y. Chuang, P.C. Chen. Prenatal smoke exposure, DNA methylation, and childhood atopic dermatitis. Clin Exp Allergy, 43 (5) (2013), pp. 535-543, [https://doi.org/10.1111/cea.12108](https://doi.org/10.4161/epi.6.11.17819) | O |
| 72 | R.M. Whyatt, W. Jedrychowski, K. Hemminki, R.M. Santella, W.Y. Tsai, K. Yang, et al. Biomarkers of polycyclic aromatic hydrocarbon-DNA damage and cigarette smoke exposures in paired maternal and newborn blood samples as a measure of differential susceptibility. Cancer Epidemiology, Biomarkers & Prevention, 10 (6) (2001), pp. 581-588. | O |
| 73 | R.M. Whyatt, R.M. Santella, W. Jedrychowski, S.J. Garte, D.A. Bell, R. Ottman, et al. Relationship between ambient air pollution and DNA damage in Polish mothers and newborns. Environ Health Perspect, 106 (Suppl 3) (1998), pp. 821-826, [https://doi.org/10.1289/ehp.98106821](https://doi.org/10.1158/1055-9965.Epi-08-0312) | O |
| 74 | C.S. Wilhelm-Benartzi, E.A. Houseman, M.A. Maccani, G.M. Poage, D.C. Koestler, S.M. Langevin, et al. In utero exposures, infant growth, and DNA methylation of repetitive elements and developmentally related genes in human placenta. Environ Health Perspect, 120 (2) (2012), pp. 296-302, <https://doi.org/10.1289/ehp.1103927> | O |
| 75 | F.Y. Wu, H.D. Wu, H.L. Yang, H.W. Kuo, J.C. Ying, C.J. Lin, et al. Associations among genetic susceptibility, DNA damage, and pregnancy outcomes of expectant mothers exposed to environmental tobacco smoke. Sci Total Environ, 386 (1-3) (2007), pp. 124-133, [https://doi.org/10.1016/j.scitotenv.2007.06.003](https://doi.org/10.1080/15592294.2017.1325065) | O |
| O – Original research paper; OP – Original research paper (retrieved in screening of projects’ outcomes); R –Review paper | | |

| **Table S2.** Search terms identified on the basis of the PECO statement | |
| --- | --- |
| **Population terms** | **antenatal**, child, **child*,** childhood, children, **fetal, fetus, fetus*,** foetus, **gestation**, ***in utero***, infant, **infant*,** **intrauterine**, **maternal**, **mother,** mother*, newborn, newborn*, **newborns**, **offspring**, **perinatal**, pregnant, **pregnan***, pregnancy, **prenatal, pre-natal**, **transgenerational**, transgenerational* |
| **Exposure terms** | **cigarette**, cigarette smok*, cigarette*, cigarettes, **cotinine**, environmental tobacco smoke, passive smok*, secondhand smok*, secondhand smoke, **smok*,** smoke, smoker, smoking, **tobacco** |
| **Outcomes terms** | alkylation, **apurinic site*, apyrimidinic site***, **chromatin remodelling**, deaminated bases, DNA adduct, **DNA adduct*,** **DNA damage*, DNA methylation, double strand break*, epigenetic, epigenetic*,** epigenetics, **genetic**, genetic damage, **genetic***, genetics, genotoxic, genotoxic*, genotoxicant, genotoxicity, **histone acetylation, histone deacetylation, histone methylation, histone modification***, histone modifications**, histone phosphorylation,** **HPRT**, **microRNA**, **miRNA**, mismatch, **mithocondrial DNA depletion**, **mutations, oxidative stress, oxidized base***, **single strand break*, sister chromatid exchange*** |

*wildcard; search terms selected to be included in tested search strings are indicated in bold

**Search string development**

To optimize the search strategy, six variations of search strings were developed and tested in PubMed. The table below summarizes the number of articles retrieved, the comprehensiveness (ability to retrieve a known set of the 75 relevant articles included in the benchmark list from Table S1), and comments on feasibility, i.e., our ability to analyze the number of retrieved results. Search strings varied in complexity, in an iterative process, combining different terms for population, exposure and outcomes from Table S2, and Boolean operators.

As shown in Table S3, the first search string (SS1) included all relevant terms using the Boolean operators ‘AND’ to combine population, and exposure groups, and ‘OR’ to pool terms within each group. This search string returned an unmanageable number of results (n=28769) considering the resources available for the preparation of this SEM, and therefore a new search string intersecting outcome terms was tested (SS2). A total number of 7840 articles were retrieved by SS2 that showed a good comprehensiveness (98.7%). Nevertheless, the number was still found excessive and, in consequence, different search strings including different terms of each group were tested for comprehensiveness, to find the best compromise between specificity and sensitivity of the search string (SS3-SS5 constitute the other examples of what was tested).

The process was stopped at SS6, when a good balance of comprehensiveness and feasibility was achieved.

| **Table S3.** Details of search strings used in search strategy refinement | | | |
| --- | --- | --- | --- |
| **ID** | **Search Terms** | **N. º articles retrieved (PubMed)** | **Comprehensiveness and Comments** |
| SS1  (Population and Exposure terms) | (tobacco OR smok* OR cigarette OR cotinine) AND (pregnan* OR gestation OR prenatal OR pre-natal OR perinatal OR antenatal OR "in utero" OR intrauterine OR fetus OR fetus* OR fetal OR offspring) | 28769 | Excessively large number of articles; not enough resources to handle this amount of information. |
| SS2  (All terms – Population, Exposure and Outcomes) | (tobacco OR smok* OR cigarette OR cotinine) AND (pregnan* OR gestation OR prenatal OR pre-natal OR perinatal OR antenatal OR "in utero" OR intrauterine OR fetus OR fetus* OR fetal OR offspring OR child* OR newborns OR maternal OR mother OR infant*) AND (genetic OR genetic* OR epigenetic OR epigenetic* OR "DNA damage" OR "single strand break*" OR "double strand break*" OR mutations OR "DNA adduct*" OR "oxidized base*" OR "oxidative stress" OR "apurinic site*" OR "apyrimidinic site*" OR "DNA methylation" OR hptr OR "histone modification*" OR "histone phosphorylation" OR "histone acetylation" OR "histone deacetylation" OR "histone methylation" OR "sister chromatid exchange*" OR "chromatin remodelling" OR "miRNA" OR microRNA OR transgenerational) | 7840 | 98.7% (74/75)  Comprehensiveness is high, but the number of retrieved articles is still excessive. |
| SS3  (Broader terms only) | (tobacco OR smok* OR smoke) AND (pregnan* OR "in utero") AND (genetic* OR epigenetic*) | 2870 | 85.3% (64/75)  Lower number of articles but also lower comprehensiveness. |
| SS4  (Broader outcome terms) | (tobacco OR smok* OR cigarette OR cotinine) AND (pregnan* OR gestation OR prenatal OR pre-natal OR perinatal OR antenatal OR "in utero" OR intrauterine OR fetus OR fetus* OR fetal OR offspring OR child* OR newborns OR maternal OR mother OR infant* OR placenta OR "cord blood") AND (genetic OR genetic* OR epigenetic) | 7079 | 84.05 (61/75) |
| SS5  (With two groups in population’ terms) | ((tobacco OR smok* OR smoke OR cigarette) AND (pregnan* OR pregnancy OR pregnant OR gestation OR "in utero" OR intrauterine OR maternal OR mother* OR prenatal OR pre-natal OR perinatal OR antenatal) AND (newborn OR offspring OR child* OR fetus OR foetus OR fetal OR infant*) AND (genetic OR genetic* OR epigenetic OR epigenetic* OR genotoxic OR genotoxic* OR cytogenetic* OR "DNA damage" OR "DNA methylation" OR "histone modification*" OR miRNA OR microRNA)) | 2885 | 92.0% (69/75) |
| SS6  (Rearranging and excluding some terms) | (tobacco OR smok* OR smoke OR cigarette) AND (pregnan* OR pregnancy OR pregnant OR gestation OR "in utero" OR intrauterine OR prenatal OR pre-natal OR perinatal OR antenatal OR ((maternal OR mother*) AND (newborn OR offspring OR child* OR fetus OR foetus OR fetal OR infant*))) AND (genetic OR genetic* OR epigenetic OR epigenetic* OR genotoxic OR genotoxic* OR cytogenetic* OR "DNA damage" OR "DNA methylation" OR "histone modification*" OR miRNA OR microRNA) | 3756 | 94.7% (71/75)  Better comprehensiveness with an acceptable number of articles. |

SS: search string

| **Table S4**. Grey literature search strategy | | |
| --- | --- | --- |
| **Search Engine** | **Search Strategy** | **Retrieved results** |
| **Google**  [www.google.com](https://doi.org/10.1289/ehp.98106821) | The most relevant terms have been selected in the following search string:  (pregnancy OR prenatal) AND (smoke OR tobacco) AND (genetic OR epigenetic) | The first 200 'hits' (not including sponsored) will be screened based on the summary description, by one reviewer, and potential relevant results will be saved using Google Chrome's Bookmark Manager, in a dedicated folder. After duplicate removal, the saved results will be screened following the same procedure used for peer-reviewed publications.  News, reports, blogs, etc. will be screened for mention/reference to other potential documents (e.g., peer-reviewed papers, thesis, prints, etc.). |
| **BASE**  [https://www.base-search.net/](https://doi.org/10.1093/hmg/ddu739) | The most relevant terms have been selected in the following search string:  (pregnancy OR prenatal) AND (smoke OR tobacco) AND (genetic OR epigenetic)  Search will be conducted in the advanced search tab, using "Verbatim search" for the Entire Document, with the "Boost open access documents" option disabled. Results will be ranked by relevance. | The results will be analysed by document type, by refining the search results with the corresponding filter. The first 50 results of each document type will be screened based on the below description, by one reviewer.  1st screening  Papers: Title + abstract  Books: Title + Description/Abstract + Chapter/Section title/Table of contents  Thesis and dissertations: Title + abstract  Reports: Title + Description/Abstract + Chapter/Section title/Table of contents  News/Newspapers/Blogs/Magazines/Websites: full text.  Potential relevant results will be saved using Bookmark Manager (Chrome) in a dedicated folder. After duplicate removal, the saved results will be screened following the same procedure used for peer-reviewed publications. News, reports, blogs, etc. will be screened for mention/reference to other potential documents (e.g.*,* peer-reviewed papers, thesis, preprints, etc.). |
| **ProQuest**  [www.proquest.com](https://doi.org/10.1002/ajmg.b.31109) | The most relevant terms were included in the following search string:  (pregnancy OR prenatal) AND (smoke OR tobacco) AND (genetic OR epigenetic) |  |

**
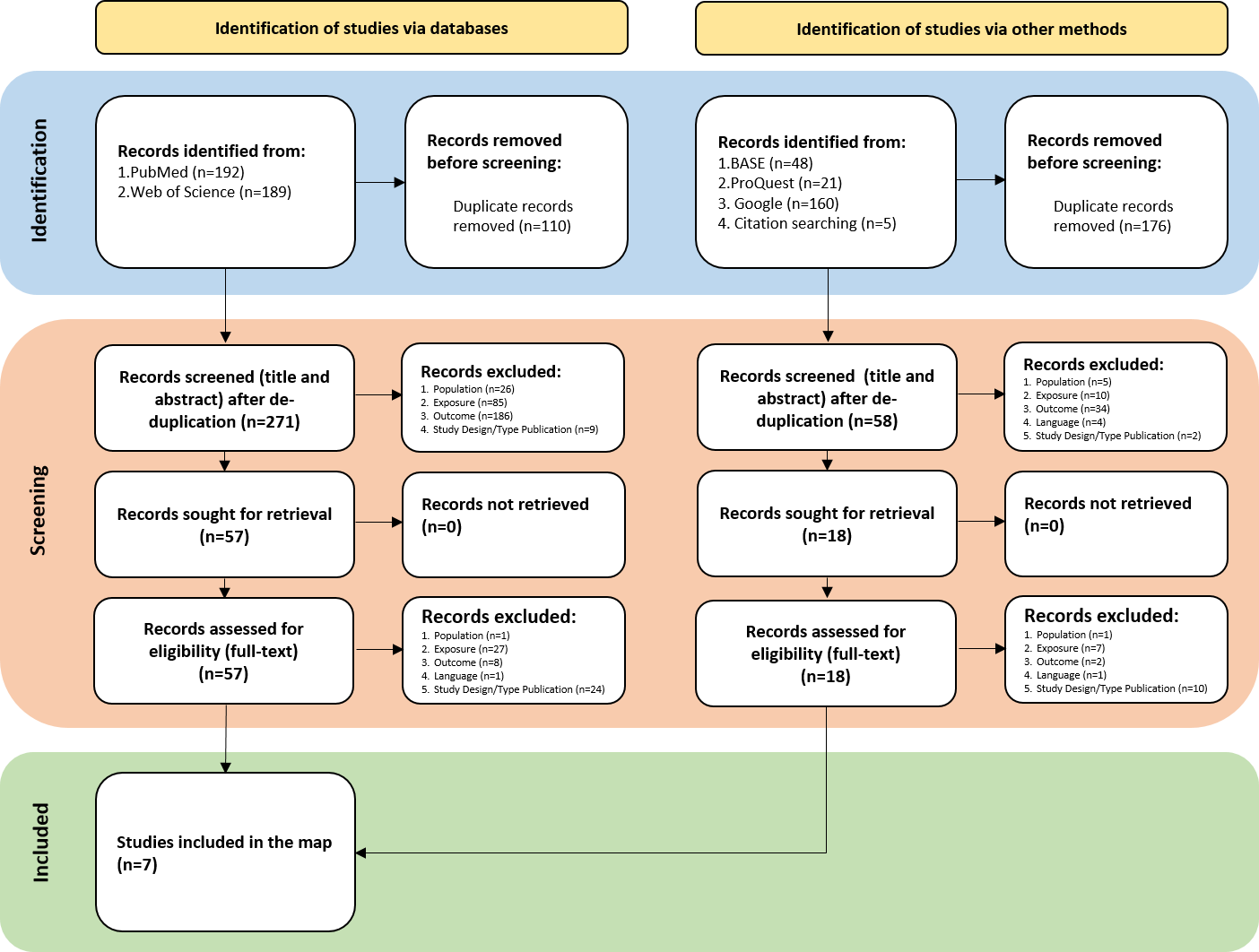
**

**Figure S1.** PRISMA flow diagram detailing the flow of information through the different stages of the pilot study
